# *Enterococcus* Phage Abitsa: Biological Characterization, Antibiofilm Activity and Evolutionary Insights into the Genus *Efquatrovirus*

**DOI:** 10.3390/v18080842

**Published:** 2026-08-01

**Authors:** Konstantin S. Troshin, Lydia I. Ilyenko, Andrei V. Chaplin, Anastasiya A. Khritova, George A. Skvortsov, Anna A. Vasilyeva, Olga Y. Guseva, Igor S. Kopetskiy, Dmitriy A. Shagin, Lyudmila I. Kafarskaia, Boris A. Efimov, Artem A. Malkov, Maxim A. Sokolovskiy, Peter V. Evseev

**Affiliations:** Pirogov Russian National Research Medical University, Ostrovityanova 1, 117997 Moscow, Russia; konstantinetr@gmail.com (K.S.T.); ilenko_li@rsmu.ru (L.I.I.); okolomedik@gmail.com (A.V.C.); nastyakhrit@yandex.ru (A.A.K.); galaxy22242500@mail.ru (G.A.S.); annavasilyeva.bioscience@gmail.com (A.A.V.); oguseva@bk.ru (O.Y.G.); kopetski@rambler.ru (I.S.K.); shagdim777@gmail.com (D.A.S.); likmed@mail.ru (L.I.K.); efimov_ba@mail.ru (B.A.E.); xkairyx@gmail.com (A.A.M.); ilasokolovskij54@gmail.com (M.A.S.)

**Keywords:** phage, *Efquatrovirus*, *Enterococcus faecalis*, *Enterococcus* phages, phage genomics, phage therapy, antimicrobial resistance, domain shuffling, phage phylogenomics

## Abstract

*Enterococcus faecalis* is an important opportunistic pathogen associated with persistent oral infections, biofilm formation, and antimicrobial tolerance, which makes phages targeting this species of both therapeutic and evolutionary interest. Here, we describe the isolation and characterization of Abitsa, a novel lytic *Enterococcus* phage representing a new species within the genus *Efquatrovirus*. Abitsa formed small plaques and displayed siphovirus-like morphology. It showed an optimal multiplicity of infection of 0.01, a short latent period of 10 min and a burst size of 28 ± 5 virions per infected cell. The phage efficiently suppressed planktonic growth of *E. faecalis* over a broad multiplicity of infection (MOI) range and significantly disrupted pre-formed biofilms, with the strongest effect observed at MOI 0.1, resulting in an 82.14% reduction in biofilm biomass. Abitsa remained stable at pH 4–8, at 5–50 °C for 1 h, and in up to 75% chloroform, but exhibited a narrow host range, lysing only one additional clinical *E. faecalis* isolate among 34 tested enterococcal isolates. Genome analysis showed that Abitsa has a 41,581-bp linear genome lacking lysogeny-associated genes and represents a novel *Efquatrovirus* species. Comparative structural and phylogenetic analyses additionally supported mosaic evolution and extensive domain shuffling in receptor-binding and lysis-related proteins of *Efquatrovirus*-like phages. Together, these results identify Abitsa as a biologically unusual and evolutionarily informative lytic phage with antibiofilm activity.

## 1. Introduction

*Enterococcus faecalis* is a Gram-positive, facultatively anaerobic coccus that normally persists as a commensal microorganism in the gastrointestinal tract of humans and animals, but can also act as an opportunistic pathogen [1]. Its pathogenic potential is associated with a broad repertoire of virulence factors, including lytic enzymes, cytolysin, aggregation substance, pheromones, and lipoteichoic acid [2], as well as with its ability to survive under adverse conditions such as extreme pH, high salinity, elevated temperature, and prolonged nutrient starvation [3]. In addition, *Enterococcus faecalis* is resistant to several antibiotics, including clindamycin, metronidazole, and aminoglycosides, which further contributes to its clinical significance [4]. The oral cavity may also serve as a reservoir of virulent *Enterococcus faecalis* strains with systemic implications. For example, this species has been identified in 60% of patients with diabetes mellitus [5] and is recognized as a potential source of life-threatening bacterial endocarditis associated with dental procedures [6].

In the oral cavity, *Enterococcus faecalis* is associated with a wide range of diseases [2,7]. It is detected in patients with caries, pulpitis, periodontitis, apical periodontitis, peri-implantitis, and other conditions, including oral cancer. *Enterococcus faecalis* is also among the bacterial species most frequently identified in root canals of teeth with post-treatment apical periodontitis, and several studies have reported an extremely high relative abundance of this microorganism in infected canals, ranging from 14% to 99% of the total microbial load [4,8]. Evidence indicates that a substantial proportion of failures in the treatment of secondary apical periodontitis are associated with infection by this microorganism. *Enterococcus faecalis* is capable of adhering to dentin and bone, penetrating dentinal tubules, and forming intraradicular and extraradicular biofilms, thereby sustaining persistent periapical lesions.

A characteristic feature of *Enterococcus faecalis* in dentistry is its tolerance to both topical and systemic antimicrobial agents. This species shows resistance to calcium hydroxide, chlorhexidine, and sodium hypochlorite, which are among the mainstays of contemporary endodontic therapy [9,10]. Literature reviews also indicate a higher prevalence of *Enterococcus faecalis* in patients with periodontitis compared with individuals with a healthy periodontium [11]. Studies of peri-implantitis have similarly demonstrated a higher prevalence of enterococci in patients with peri-implant inflammation [12]. In addition, an increasing number of studies suggest that *Enterococcus faecalis* may contribute to oral carcinogenesis. In particular, it may promote proliferation of oral cancer cells via an epidermal growth factor receptor signaling pathway mediated by H_2_O_2_ [13], and its pathogenic effects may be further enhanced in combination with other opportunistic microorganisms such as *Candida albicans* [14]. Given the challenges of treating apical periodontitis and the association of *Enterococcus faecalis* with multiple oral pathologies, identifying effective strategies to eliminate this microorganism from the oral cavity is highly relevant.

Bacteriophages (phages), viruses that infect bacterial cells, are of interest both as convenient models for genomic and evolutionary studies and as promising agents for the treatment of antibiotic-resistant infections. Tailed phages classified within the class *Caudoviricetes* are of particular interest in evolutionary research because their genomes are highly modular, and their multidomain proteins can undergo domain shuffling. These properties make them useful models for tracking the formation of chimeric viral genomes and proteins [15,16,17].

Bacteriophage therapy also represents a promising approach for the treatment of *Enterococcus faecalis* infections, including in combination with antibacterial agents [18]. Phages targeting *Enterococcus faecalis* have shown efficacy in experimental infection models and have also been applied in individual clinical cases, indicating the broader therapeutic potential of this approach [19,20,21]. In murine experiments, the administration of a single intraperitoneal injection of the *E. faecalis* phage EF-P29 at 1 h after challenge was sufficient to protect all mice against bacteremia caused by infection with a vancomycin-resistant *Enterococcus faecalis* strain [20]. Experiments conducted by Gelman et al. [22] showed that one injection of a bacteriophage cocktail was sufficient to significantly reduce mortality caused by vancomycin-resistant *Enterococcus faecalis* in a mouse model.

Because of the major role of *Enterococcus faecalis* in persistent endodontic infections, phage-based approaches have attracted particular interest in dentistry [23,24]. Recent studies have demonstrated effective biofilm disruption using phages. Phage SHEF2, isolated from the mouth of a patient with an infected root canal, eradicated biofilm formed on polystyrene in vitro and resolved *Enterococcus faecalis* infection in a zebrafish model system [25]. The combination of phage and NaOCl applied on dentin discs resulted in an 84% reduction in biofilm mass [26]. In a human root canal model, irrigation with the lytic phage EFDG1 reduced bacterial leakage from the root apex compared with the control group, while viability of *Enterococcus faecalis* decreased by approximately 7 log units after phage irrigation [27].

In this study, a new lytic phage, Abitsa, was isolated from the Bitsa River in the Moscow region, Russia. Its morphology, biological properties, and antibiofilm activity were characterized. Particular attention was paid to the characterization of the phage genome and proteins.

## 2. Materials and Methods

### 2.1. Phage Isolation and Purification

*Enterococcus faecalis* ATCC 19433, a commercial reference commensal-type strain, was used as the host strain for phage isolation and propagation. The sequence type (ST) of this strain was determined in silico using the MLST software (v2.23.0) with default parameters (https://github.com/tseemann/mlst, accessed on 20 March 2026). For phage enrichment, 20 mL of a water sample collected from the Bitsa River (Moscow Region, Russia) was centrifuged at 8000× *g* for 15 min (Nuve NF 1200 R, NÜVE, Ankara, Turkey), and the obtained supernatant was filtered through a membrane filter with a pore size of 0.22 μm (Guangzhou Jet Bio-Filtration Co., Ltd., Guangzhou, China). The filtered sample was supplemented with 10 mL of 3× brain heart infusion (BHI) broth (Becton-Dickinson, Franklin Lakes, NJ, USA) and 100 µL of an overnight culture of *Enterococcus faecalis* ATCC 19433. After incubation for 24 h at 37 °C with shaking, chloroform (Aldosa, Moscow, Russia) was added to a final concentration of 5% (*v*/*v*), and the mixture was incubated for 15 min at 37 °C, followed by centrifugation at 10,000× *g*. The presence of phages was confirmed using the double-agar layer technique [28], with a soft overlay containing 0.7% agar. Serial dilutions of the phage-containing supernatant were plated on a lawn of *Enterococcus faecalis* ATCC 19433, and a single plaque was transferred into 950 µL of SM buffer (50 mM Tris-HCl, 100 mM NaCl, 8 mM MgSO_4_, pH 7.5), treated with 50 µL of chloroform, and centrifuged as described above. To propagate the phage from a single plaque, 40 mL of an overnight culture of *Enterococcus faecalis* ATCC 19433 was centrifuged at 8000× *g* for 15 min, resuspended in 10 mL of BHI broth supplemented with 100 µL of 1 M MgCl2, and infected at a multiplicity of infection (MOI) of 0.01 with the phage lysate obtained from the single plaque. This mixture was incubated for 30 min at 37 °C and then added to 400 mL of BHI broth and incubated for 24 h with shaking. The resulting phage lysate was precipitated with PEG 8000 (10%) (CDH, Daryaganj, India) and NaCl (1 M), followed by centrifugation at 8000× *g*, resuspension of the pellet in SM buffer, and an additional washing step that included centrifugation at 13,000× *g* for 90 min, the obtained pellet was resuspended in SM buffer. The resulting phage stock was stored at 4 °C until use.

### 2.2. Transmission Electron Microscopy

Phage samples were prepared for negative-stain TEM on Formvar-coated copper grids. Briefly, approximately 200 µL drops of phage suspension in 0.1 M ammonium acetate were placed on a sheet of Parafilm M (Bemis Company, Inc., Neenah, WI, USA). Copper TEM grids (200–400 mesh, Formvar-coated) were floated on the droplets with the film side facing down and allowed to adsorb for approximately 2 min at room temperature. Excess liquid was removed by touching the edge of each grid to filter paper. The grids were then stained with 1.5% (*w*/*v*) uranyl acetate (Electron Microscopy Sciences, Morgantown, PA, USA) for approximately 1 min, blotted with filter paper, and air-dried. Samples were examined using a JEM-1011 transmission electron microscope (JEOL Ltd., Tokyo, Japan) operated at 80 kV under standard conditions. Capsid and tail sizes of phages were measured using ImageJ v. 1.54g (National institutes of health, Bethesda, MD, USA).

### 2.3. Optimal Multiplicity of Infection and In Vitro Bacterial Lytic Efficiency

The optimal multiplicity of infection, defined as the ratio of infectious phage particles to host bacteria at the time of infection, was determined as follows. *Enterococcus faecalis* cells at a concentration of 10^7^ CFU/mL were mixed with phage Abitsa at varying concentrations to achieve target MOI values of 10, 1, 0.1, 0.01, and 0.001. The mixtures were incubated overnight at 37 °C with shaking at 180 rpm [29]. After incubation, the cultures were diluted to an appropriate concentration with 0.9% NaCl solution, and the phage titer was determined using the double-layer plaque assay. The MOI that yielded the highest phage titer was defined as the optimal MOI. This experiment was performed in three independent replicates to ensure the reliability and reproducibility of the results.

The effects of phage Abitsa on bacterial growth in liquid culture were evaluated using a dynamic growth inhibition assay [30]. The experiment was conducted at MOIs of 10, 1, 0.1, 0.01, and 0.001. Each well contained 100 μL of BHI broth, 100 μL of a mid-log-phase *Enterococcus faecalis* suspension (10^7^ CFU/mL), and 100 μL of an appropriately diluted phage suspension (10^8^–10^4^ PFU/mL). In the untreated bacterial control, the phage suspension was replaced with an equal volume of BHI broth. The plate was incubated at 37 °C with continuous shaking in a microplate reader (Hangzhou Miu Instruments Co., Ltd., Hangzhou, China), and OD600 was recorded every 30 min for 12 h. The experiment was performed in three independent biological replicates.

To detect phage-resistant bacteria, aliquots were collected from phage-treated cultures at 2, 4, 6, 8, 10, and 12 h and plated on BHI agar to obtain individual colonies. Three colonies from each MOI and time point in each biological replicate were tested for susceptibility to phage Abitsa using the double-layer agar method. A 30 μL aliquot of phage suspension at 10^6^ PFU/mL was applied to each bacterial lawn. The parental *E. faecalis* ATCC 19433 strain was included as a phage-susceptible control. Colonies showing no visible lysis zone after 24 h at 37 °C were classified as phage-resistant under the tested conditions. The experiment was performed in three independent biological replicates.

### 2.4. Testing of Antibiofilm Activity of the Phage

A mature biofilm eradication assay was performed to evaluate the activity of phage Abitsa [29,31]. First, 100 μL of a mid-log-phase *Enterococcus faecalis* suspension (1 × 10^7^ CFU/mL) was inoculated into a 96-well plate (Wuxi Nest Biotechnology Co., Ltd., Wuxi, China) and incubated statically at 37 °C for 24 h to allow mature biofilm formation. Subsequently, 100 μL of phage suspensions at concentrations of 1 × 10^8^, 1 × 10^7^, 1 × 10^6^, 1 × 10^5^, 1 × 10^4^ PFU/mL were added to achieve target MOI values of 10, 1, 0.1, 0.01, and 0.001, calculated relative to the initial bacterial inoculum, and followed by an additional 24 h incubation. A control well containing BHI broth without phage was included. After incubation, planktonic cells were removed by gently washing each well three times with 300 μL of 0.9% NaCl solution. The adherent biofilms were then fixed with 200 μL of methanol for 30 min and stained with 200 μL of 0.1% (*w*/*v*) crystal violet solution for 20 min. Unbound dye was removed by washing the wells three times with 0.9% NaCl solution. Finally, the bound crystal violet was solubilized with 250 μL of 33% (*v*/*v*) acetic acid, and the absorbance was measured at 570 nm using a microplate reader (Hangzhou Miu Instruments Co., Ltd., Hangzhou, China). All assays were performed in three biological replicates to ensure statistical reliability.

### 2.5. Phage One-Step Growth Curve Assay

To determine the single-cycle burst size and latent period, a 15 mL culture of *Enterococcus faecalis* ATCC 19433 at OD600 ~0.4 was centrifuged at 8000× *g*, resuspended in 0.5 mL of BHI, infected with 100 µL of phage suspension (1.5 × 10^8^ PFU/mL) at an MOI of 0.01, and incubated for 5 min at 37 °C. Unbound phages were removed by centrifugation for 2 min at 12,000× *g*, the supernatant was removed, and the pellet was resuspended in 10 mL of BHI. The suspension was incubated in 15 mL tubes at 37 °C on a shaker (ES-20 orbital shaker, Biosan, Riga, Latvia) for 90 min at 200 rpm, with 100 µL samples collected at the start time (t0) and at 10 min intervals up to 90 min from the start of the experiment. The experiment was performed in three biological replicates. For PFU/mL determination, the samples were titrated, and 10 µL aliquots were spotted onto double-layer BHI agar, followed by plaque counting after 24 h of incubation. Phage burst size was calculated as the ratio of the average titer at the plateau after the burst to the total number of phages at t0 [32].

### 2.6. Phage Stability Under Different Conditions

Phage stability under various environmental conditions was assessed using a protocol based on [33] with modifications. To determine temperature stability, the phage lysate was diluted tenfold in SM buffer to a final concentration of 5.5 × 10^6^ PFU/mL. Aliquots (500 µL) of this suspension were then incubated at 5 °C, 25 °C, 37 °C, 50 °C, 60 °C, and 70 °C for 1 h using a TDB-20 dry block thermostat (Biosan, Riga, Latvia). To assess pH stability, SM buffer was adjusted with NaOH or HCl to pH values ranging from 2 to 12. These solutions were added to the phage samples to obtain a final phage titer of approximately 5.5 × 10^6^ PFU/mL, and the mixtures were incubated at 37 °C for 1 h. To determine UV resistance, 500 µL aliquots of phage lysate in 1.5 mL Eppendorf polypropylene tubes were placed at a distance of 60 cm from a TUV 30 W/G30 T8 UV lamp (Philips, Amsterdam, The Netherlands) emitting at 254 nm for 10 min. Aliquots of 50 µL were collected before the start of irradiation and after 30 s, 1, 2, 3, 4, 5, and 10 min of exposure. Chloroform sensitivity was evaluated by mixing phage solutions in SM buffer with chloroform to achieve final concentrations of 0%, 5%, 25%, 50%, and 75% (*v*/*v*), resulting in a final titer of 3.3 × 10^5^ PFU/mL, and the mixtures were incubated at 37 °C for 1 h according to [34]. Subsequently, the solutions were centrifuged at 10,000× *g* for 10 min, and the supernatant was collected. All resulting samples were titrated and plated using the double-layer agar method. After 24 h of incubation, the titer was determined. All experiments were performed in three biological replicates.

### 2.7. Phage Host Range Determination

The phage host range was evaluated against 26 clinical isolates of *Enterococcus faecalis* and 8 clinical isolates of *Enterococcus faecium* using the double-layer agar assay. Overnight cultures of *Enterococcus faecalis* and *Enterococcus faecium* (200 μL) were mixed with 3 mL of 0.7% BHI agar and plated. Phage suspensions (10^7^ PFU/mL) were then spotted onto the bacterial lawns and incubated at 37 °C for 18–24 h. The plates were subsequently examined for plaque formation.

### 2.8. Statistical Analysis

The obtained data were analyzed statistically using GraphPad Prism 10.5.0 (GraphPad Software, Boston, MA, USA). Differences among the tested conditions for each parameter were assessed using one-way ANOVA followed by Tukey’s HSD post hoc test [35]. A value of *p* ≤ 0.05 was considered statistically significant.

### 2.9. Genome Assembly and Annotation

Phage DNA was isolated by phenol–chloroform extraction and subsequently fragmented using a Bioruptor sonicator (Diagenode, Liège, Belgium). The genome was assembled using Unicycler v0.5.1 [36]. Phage termini were determined using PhageTerm 1.0.12 available on the Galaxy Pasteur server [37]. Coding sequences were identified using Pharokka 1.7.5 [38]. Functional annotation of the predicted genes was performed using a combination of HMM-HMM comparison and structure-based searches. HHpred searches were performed against the PDB70_mmCIF70_30_Mar, PfamA-v37, UniProt-SwissProt-viral70_3_Nov_2021, and NCBI_Conserved_Domains (CD)_v3.19 databases [39]. Foldseek and Dali searches [40,41] were performed using structures predicted by AlphaFold 3 [42], and key structural predictions were additionally verified using Chai-1 [43].

### 2.10. Domain Shuffling Analysis

For the analysis of domain shuffling within the genus *Efquatrovirus*, all genome sequences from the core_nt database showing >65% BLASTn identity over >65% query coverage were downloaded from NCBI GenBank (https://blast.ncbi.nlm.nih.gov/Blast.cgi, accessed on 26 February 2026), excluding uncultured samples and phages missing large essential genome fragments; the final dataset included 91 *Efquatrovirus* genomes. Orthologous groups were constructed for the encoded proteins using PIRATE v1.0.5 [44], sequences within these groups were hierarchically clustered using FastTree 2.1.11 [45], and structures of representative cluster members were predicted using AlphaFold 3 [42].

### 2.11. Intergenomic Similarity Comparisons and Phylogenetic Analysis

Phage genomes were retrieved from the GBPHG GenBank dataset (https://ftp.ncbi.nlm.nih.gov/genbank, accessed on 20 March 2026). Only records explicitly annotated as complete genome were retained. Intergenomic similarities of phages were calculated using VIRIDIC applying default settings [46].

Phylogenetic analysis was performed as follows. Genomes related to *Enterococcus* phage Abitsa were identified using the Abitsa major capsid protein (MCP) and terminase large subunit (TLS) as queries. Homology searches were conducted with an E-value cutoff of 0.001, and only genomes recovered in both MCP- and TLS-based searches were retained. To standardize gene calling, proteins in all selected genomes were re-predicted with Prodigal v2.6.3 in single-genome mode, and the best MCP- and TLS-like homologs were identified by BLASTp against the Prodigal-derived protein sets.

Only taxa represented by binomial names were treated as ICTV-classified. Taxonomic labels lacking binomial names were treated as NCBI-annotated, whereas records lacking taxonomy or annotated only as *Caudoviricetes* were treated as unclassified. The final comparative dataset for MCP- and TLS-based phylogenies was assembled using a combined taxonomic and phylogenetic framework. The focal block consisted of six phages, namely *Enterococcus* phage Abitsa and five ICTV-classified *Efquatrovirus* phages. For all remaining taxon groups, one representative per group was selected, preferentially from ICTV-classified taxa. Additional NCBI-annotated and unclassified phages were included to represent neighboring and more distant clades relevant to the evolutionary placement of Abitsa and *Efquatrovirus*. To avoid overrepresentation of large clusters of highly similar phages, particularly unclassified *Efquatrovirus*-like enterococcal phages, candidate additions were constrained using genetic-distance criteria derived from both MCP and TLS phylogenies. This procedure yielded the final 50-phage dataset.

Protein sequences were aligned with MAFFT v7.526 using the L-INS-i algorithm (--localpair --maxiterate 1000). Maximum-likelihood trees were inferred with IQ-TREE v3.0.1 using ModelFinder, SH-aLRT support with 1000 replicates, and ultrafast bootstrap with 1000 replicates. Root positions for the final MCP, TLS, and portal protein trees were additionally evaluated using the non-reversible amino acid substitution model implemented in IQ-TREE with the --model-joint NONREV option.

In addition to MCP and TLS, the following proteins were analyzed: DNA helicase (HEL), DNA polymerase (DNAP), distal tail protein (DTP), endolysin (EL), major tail protein (MTP; tail tube protein), portal protein (PP), and tail tip protein (Tal). Homologs of these proteins were first identified within the final 50-phage MCP/TLS dataset, retaining the best hit per genome. Because some of these proteins were absent from part of the initial dataset, the HEL, DNAP, DTP, EL, MTP, and Tal datasets were then expanded to 50 sequences each by searching the complete GBPHG protein database, excluding phages already present in the original MCP/TLS candidate pool. All taxa already present in the preliminary trees were retained. Additional sequences were incorporated in the following order of priority: the single closest new homolog regardless of annotation status, followed by additional ICTV-classified, NCBI-annotated, and unclassified candidates. To limit redundancy, no more than one newly added representative per taxon group was retained whenever possible, again with preference given to ICTV-classified taxa. Highly similar candidates were filtered using a 50% pairwise similarity threshold, unless this had to be relaxed to reach 50 taxa. Expanded datasets were aligned with MAFFT L-INS-i and analyzed with IQ-TREE using the same settings as described above.

## 3. Results

### 3.1. Phage Morphology

**Figure 1:**
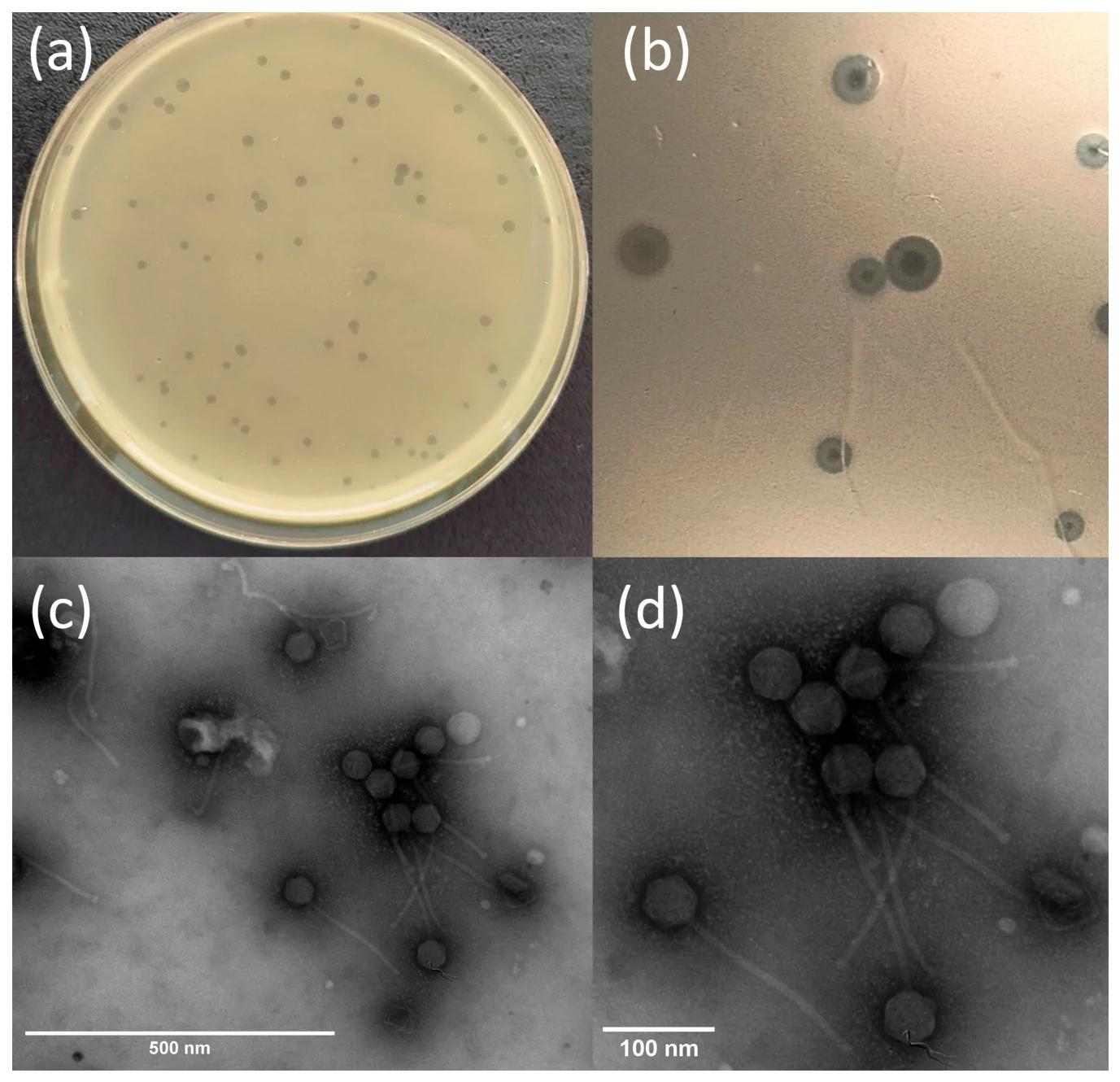
(**a**,**b**) Abitsa plaques on double layer BHI agar. (**c**,**d**) Two representative electron microscopic images of phage Abitsa particles.

Abitsa formed small circular plaques 1–2 mm in diameter with a clear central zone and a turbid surrounding ring on double-layer agar after 24 h of incubation (Figure 1a,b).

Transmission electron microscopy revealed that phage Abitsa exhibited a morphology characteristic of siphoviruses, with an icosahedral capsid of 44 ± 2 nm in diameter measured from facet to facet and a long flexible tail of 167 ± 9 nm in length according to measurements of ten phages. (Figure 1c,d).

### 3.2. Optimal Multiplicity of Infection and Phage Kinetics in Planktonic Culture

**Figure 2:**
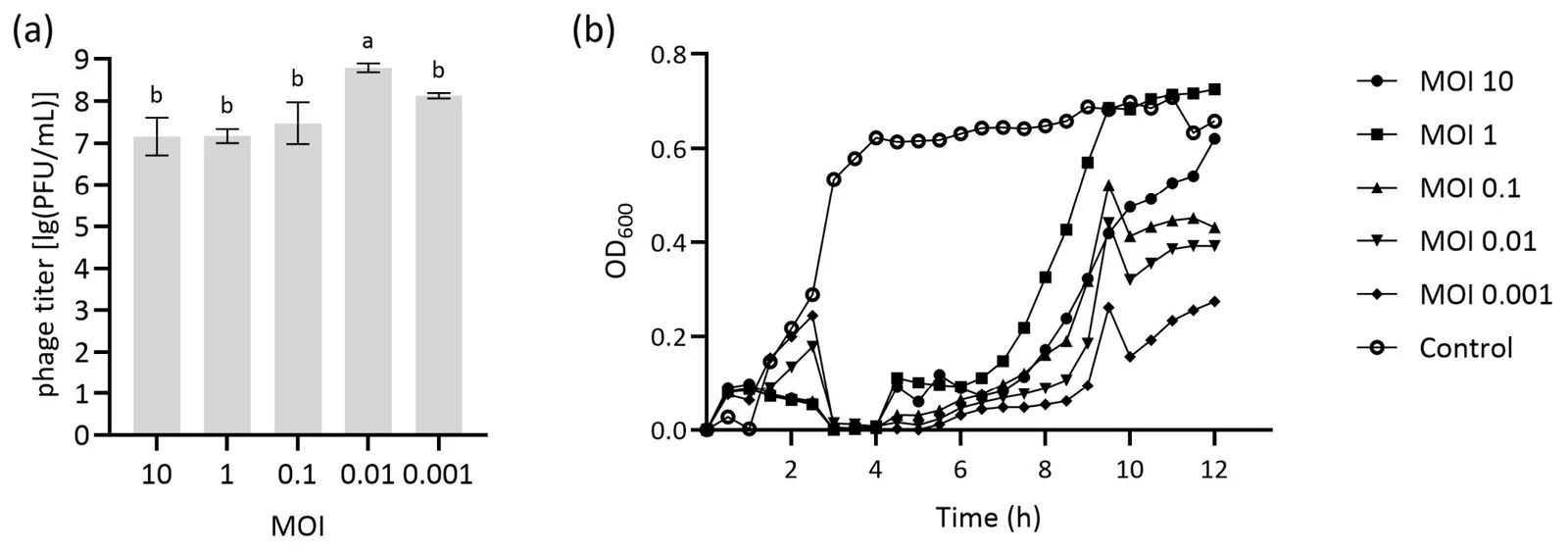
(**a**) Optimal multiplicity of infection (MOI) determination. All values represent the mean ± SD of three independent trials. Different lowercase letters above columns indicate statistically significant differences between groups as determined by Tukey’s HSD test (*p* < 0.05) following a significant one-way ANOVA. (**b**) Evaluation of the in vitro antibacterial activity of phage Abitsa. Time–bacteriostatic dynamics curves.

In the MOI experiment, the highest progeny phage titer of Abitsa was obtained at an MOI of 0.01, reaching 6.33 × 10^8^ PFU/mL after 24 h (*p* < 0.05). Thus, the optimal MOI of phage Abitsa was determined to be 0.01 (Figure 2a).

Bacterial growth curves obtained at each tested MOI are shown in Figure 2b. The OD600 profiles demonstrated two distinct phases: an initial period of bacterial growth suppression followed by regrowth. During the first 2 h, cultures treated at MOIs of 10, 1, and 0.1 maintained relatively low OD600 values, whereas cultures treated at MOIs of 0.01 and 0.001 showed a transient increase in OD600. Subsequently, OD600 decreased to near-baseline levels at approximately 3–4 h in all phage-treated cultures, consistent with phage-mediated growth suppression and lysis (Figure 2b).

After approximately 4 h, OD600 increased again in all phage-treated cultures. The timing and magnitude of this regrowth did not show a simple monotonic relationship with the initial MOI. The culture treated at an MOI of 1 showed pronounced regrowth and reached an OD600 close to that of the untreated control by the end of the experiment. At an MOI of 10, regrowth occurred later and remained less pronounced than at an MOI of 1. Cultures treated at MOIs of 0.1, 0.01, and 0.001 showed lower final OD600 values in the growth curves.

To determine whether regrowth was associated with phage resistance, colonies recovered at successive time points were tested for susceptibility to phage Abitsa. At 2 h, all tested colonies remained susceptible to the phage. Phage-resistant colonies were recovered from 4 h onward at MOIs of 10, 1, 0.1, and 0.01 and remained detectable through 12 h. At an MOI of 0.001, resistant colonies were not detected at 4 h but were recovered from 6 h onward. These findings indicate that the later increase in OD600 was associated with the outgrowth of phage-resistant bacterial variants.

### 3.3. Phage Efficacy Against Pre-Formed Biofilms

The antibiofilm activity of bacteriophage Abitsa against *Enterococcus faecalis* was evaluated by measuring optical density at 570 nm (OD570). Lower OD570 values relative to the control indicated a reduction in biofilm biomass or metabolic activity. Statistical significance among groups was assessed using one-way ANOVA (*p* = 0.005) followed by Tukey’s post hoc test.

**Figure 3:**
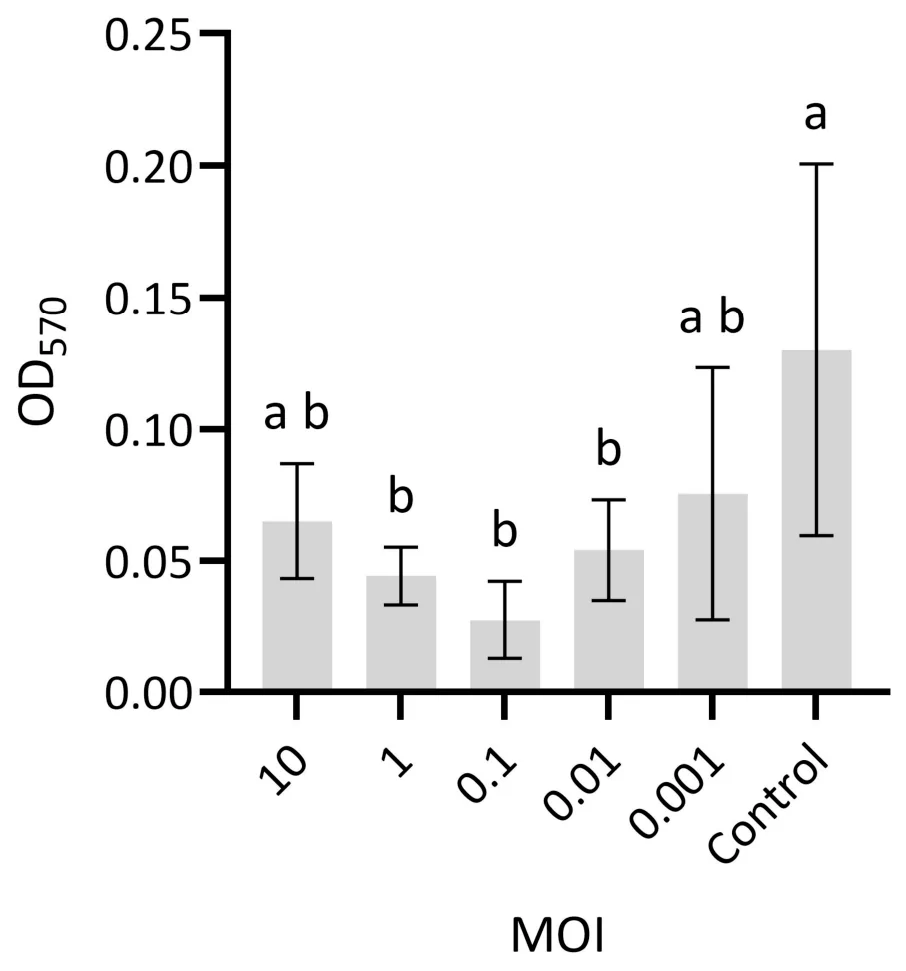
Mature biofilm eradication: Evaluation of residual 24 h biofilm biomass using crystal violet staining. All values represent the mean ± SD of three independent trials. Different lowercase letters above columns indicate statistically significant differences between groups (Tukey’s HSD test, *p* < 0.05), whereas columns sharing the same letter are not significantly different from each other.

Significant biofilm disruption was observed at lower MOIs. The strongest effect was detected at an MOI of 0.1, which resulted in an 82.14% reduction in biofilm biomass. The second strongest effect was observed at an MOI of 1, with a 68.35% reduction compared with the untreated control (Figure 3). The untreated control group maintained stable and relatively high OD570 values, confirming robust biofilm formation in the absence of phage treatment.

### 3.4. One-Step Curve and Phage Stability Under Different Conditions

**Figure 4:**
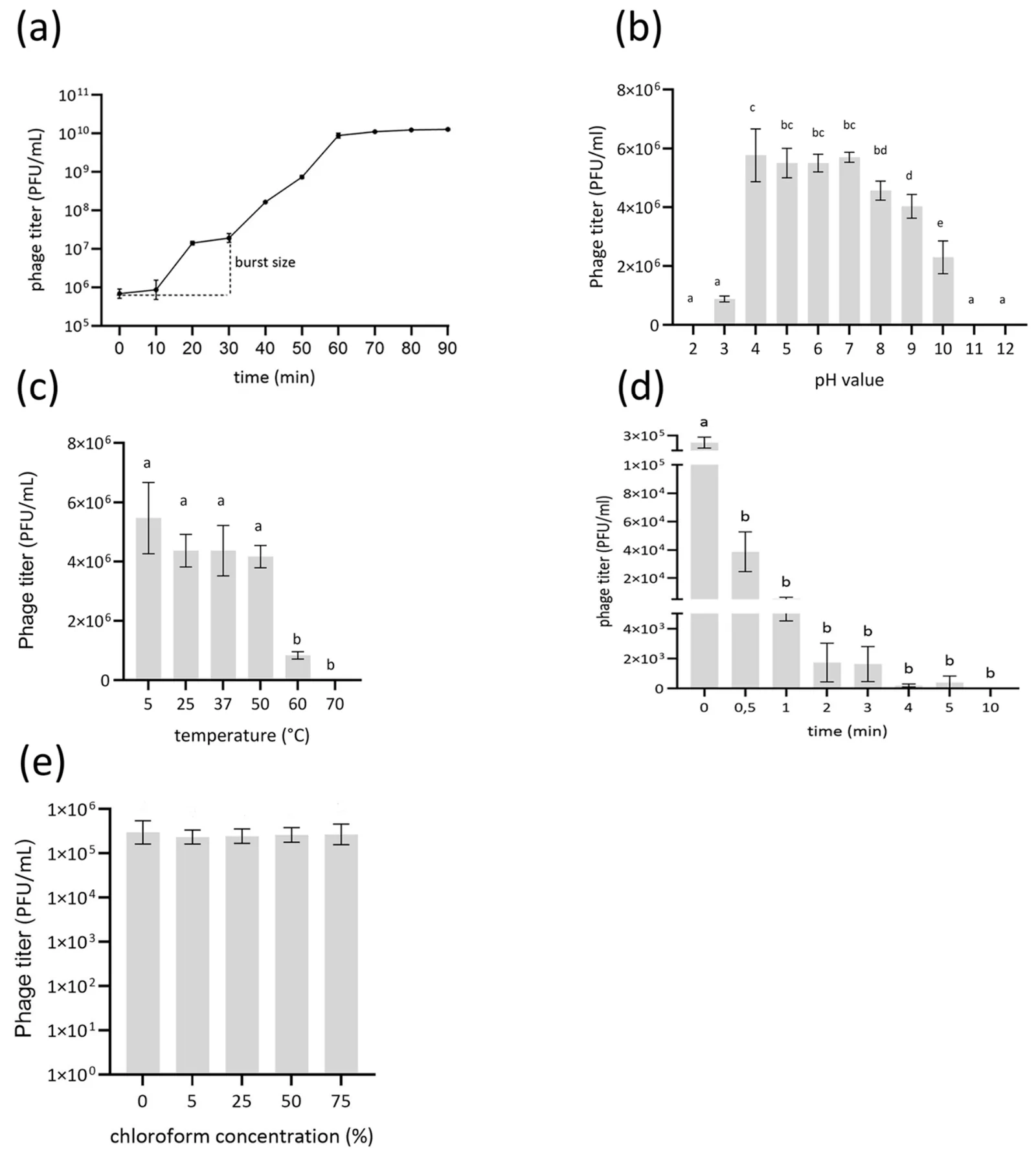
(**a**) Phage growth curve. (**b**) pH stability. (**c**) Thermal stability. (**d**) UV irradiation stability. (**e**) Stability in chloroform. All values represent the mean ± SD of three independent trials. Statistical analysis (Panels **b**–**d**): Different lowercase letters above columns indicate statistically significant differences between groups as determined by Tukey’s HSD test (*p* < 0.05) following a significant one-way ANOVA. (**e**) Chloroform stability: No significant differences were found (one-way ANOVA, *p* = 0.89).

Based on the one-step growth curve, phage Abitsa had a latent period of 10 min, followed by a 10 min rise period, and reached the first plateau at 20 min after the start of the experiment. After reaching the first plateau, the phage titer continued to increase, and a second plateau was reached at 70 min with a titer of 1 × 10^10^ PFU/mL, indicating that multiple bursts had occurred (Figure 4a). The burst size, calculated as the ratio of the average titer at the first plateau after the burst to the total number of phages at t0, was 28 ± 5 phages.

The optimal pH range for phage Abitsa was 4–8; exposure to pH 2–3 and pH 9–12 resulted in a significant partial or complete loss of viability (*p* < 0.05) (Figure 4b). Abitsa remained stable at 5–50 °C after 1 h of incubation, whereas a significant decrease in titer was observed after 1 h at 60 °C, and no plaques were detected on double-layer agar after incubation at 70 °C (Figure 4c). Exposure to UV irradiation reduced the phage titer by 80% after 30 s and by 99.9% after 4 min, while complete inactivation occurred after 10 min (Figure 4d). The phage also showed exceptionally high stability in chloroform, with no statistically significant differences (*p* = 0.89) in titer even after exposure to 75% chloroform for 1 h (Figure 4e).

### 3.5. Phage Host Range

The *E. faecalis* ATCC 19433 strain used for the isolation of phage Abitsa, is a reference strain whose sequence type was determined to be ST25. This is a non-resistant, non-capsulated strain with a 14.1% smaller genome than the well characterized *Enterococcus faecalis* V583, sharing the same auxotrophies and core metabolism with V583 [47]. Genomic analysis confirmed the presence of the PIP (phage infection protein) in this strain, which is known to serve as a receptor for some lytic phages. The EPA cluster of *E. faecalis* ATCC 19433 was found to carry epaA (locus tag EF2198), epaE (EF2194), epaM (EF2182), epaN (EF2181) and epaR (EF2177), whereas epaB (EF2197) was not detected. The phage host range was evaluated against a total of 33 *E. faecalis* and *E. faecium* clinical isolates in addition to the host strain used for isolation. Abitsa host range was found to be limited, with lytic activity against only one additional *E. faecalis* clinical isolate (Table 1).

### 3.6. Genome General Characterization

**Figure 5:**
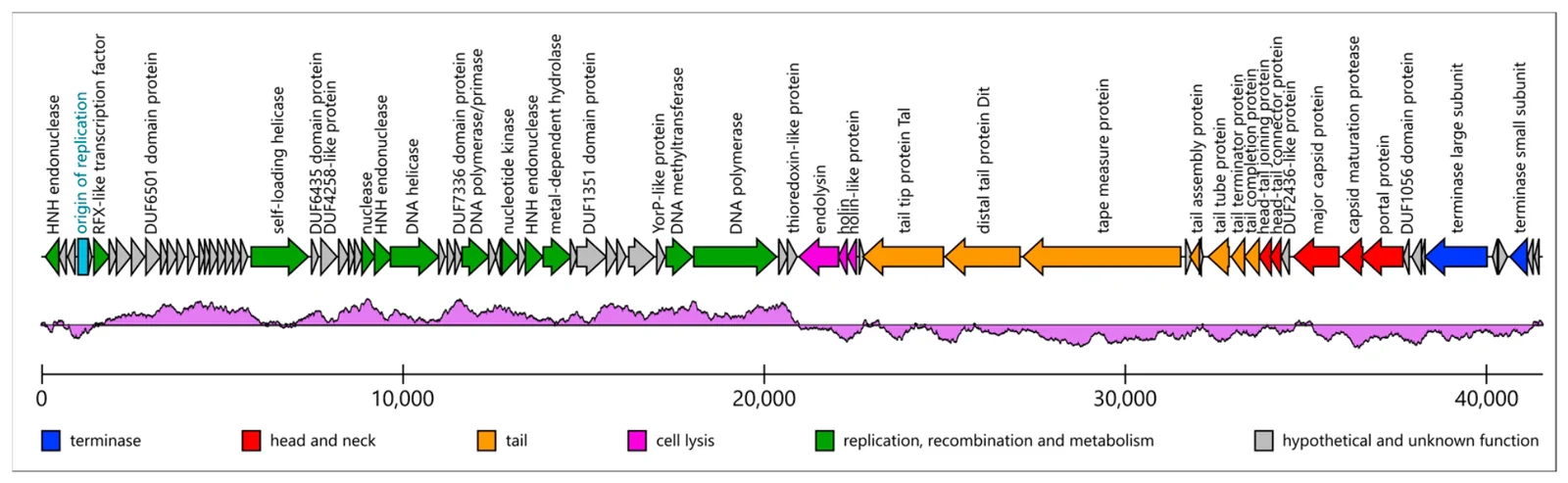
Genome map of the phage Abitsa. Coding sequences are colored based on their general functions, arrows directions represent the direction of transcription. Graph under the genome map represents GC-skew calculated using sliding window of 500 bp.

The genome of phage Abitsa consists of a linear DNA molecule of 41,581 bp with a GC content of 34.66%. PhageTerm analysis revealed defined genome ends with putative 3′ overhangs, similar to those reported for phage HK97. This finding is consistent with a previous report on the related *Efquatrovirus* phage IME-EF4 [48]. The genome consists of two halves with different predominant directions of transcription and distinct GC-skew patterns (Figure 5). One half represents a putative replication module, whereas the other contains consecutive modules encoding DNA packaging, head, tail, and lysis proteins. The genome does not encode integrases, lysogeny-associated repressors, or MuA-like transposases, suggesting an obligately lytic and non-integrating lifestyle.

**Figure 6:**
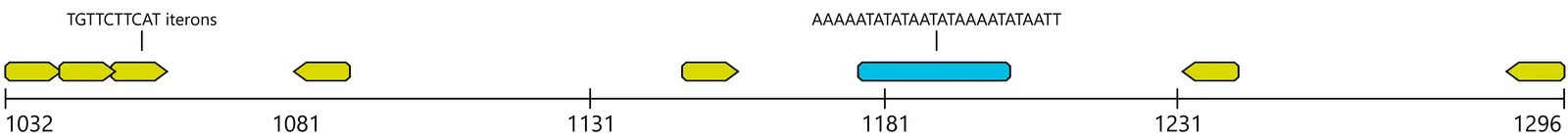
Putative replication origin of phage Abitsa genome. Coordinates represent positions in the whole genome sequence.

GC-skew and gene transcription directions suggest the presence of a single origin of bidirectional replication near one of the genome termini (Figure 5). Replication is putatively initiated by the self-loading helicase Abitsa_023, which is homologous to the helicase region of PriRep5 encoded by the *Staphylococcus aureus* pathogenicity island SaPI5 [49]. We therefore searched phage Abitsa for a replication origin architecture similar to that of SaPI5 [50] and identified a set of seven 10 bp repeats (iterons) surrounding an AT-rich region at the expected genomic locus (Figure 6).

The replication module additionally contains a large set of putative non-structural accessory genes that may facilitate host metabolic reprogramming or help overcome phage defense systems. The predicted nucleotide kinase Abitsa_038, whose C-terminal half is similar to gp1.7 of *Escherichia* phage T7 [51], may provide a supply of dNTPs to support phage genome replication. A DNA-(adenine-N6)-methyltransferase (Abitsa_048) is related to Dam methyltransferases of *Escherichia coli* and *Escherichia* phage T4 and may protect the phage genome from cleavage by host endonucleases [52] or other methylation-sensing systems such as BREX [53]. A metal-dependent hydrolase (Abitsa_041) has an MβL superfamily fold and contains the conserved HxHxDH motif involved in binding two Zn2+ ions. It also possesses a positively charged surface near the active site, similar to that observed in the predicted structure of the exodeoxyribonuclease YycJ [54], suggesting binding of a large nucleic acid molecule as a substrate and a possible nuclease activity.

The lysis module of phage Abitsa differs from the canonical holin–endolysin pair. It contains two holin proteins, Abitsa_054 and Abitsa_053, which are homologous to proteins 24.1 and 26 of phage SPP1, respectively. The peptidoglycan-degrading enzyme of phage Abitsa (Abitsa_052) contains a signal peptide and therefore belongs to the exported endolysins (e-endolysins). Its N-terminal catalytic domain (residues 26–197) belongs to the Pfam Amidase_2 family and is predicted to cleave the amide bond between N-acetylmuramic acid and L-alanine. The small middle domain (residues 209–280) adopts an SH3-like fold, although its function remains unknown. The C-terminal domain (residues 290–365) is highly similar to the lysostaphin SH3b domain (PDB 7bng; RMSD 1.21 over 96% query coverage) and is therefore predicted to bind the branched peptide stems of *Enterococcus* peptidoglycan.

### 3.7. Structural Proteins

We did not identify a separate scaffolding protein gene in the Abitsa genome. Instead, the encoded major capsid protein (Abitsa_068) contains a long N-terminal region (residues 1–177) with a poorly predicted structure and a high electrostatic charge (27.7% glutamate and 15.3% lysine residues). We suggest that this region may represent a scaffolding domain connected to the N-arm. The remaining part of the protein has the canonical fold consisting of the E-loop, P-domain, and A-domain. According to a DALI search, the closest experimentally determined related structures are those of the capsid-forming phage-inducible chromosomal island EcCI2 (PDB 9gyi) and phage HK97 (PDB 1ohg). Both proteins also harbor a scaffolding region that is cleaved during capsid assembly [55,56]. The neighboring gene encodes a capsid maturation protease (Abitsa_069), assigned to family S78 according to BLASTp against the MEROPS database [57].

**Figure 7:**
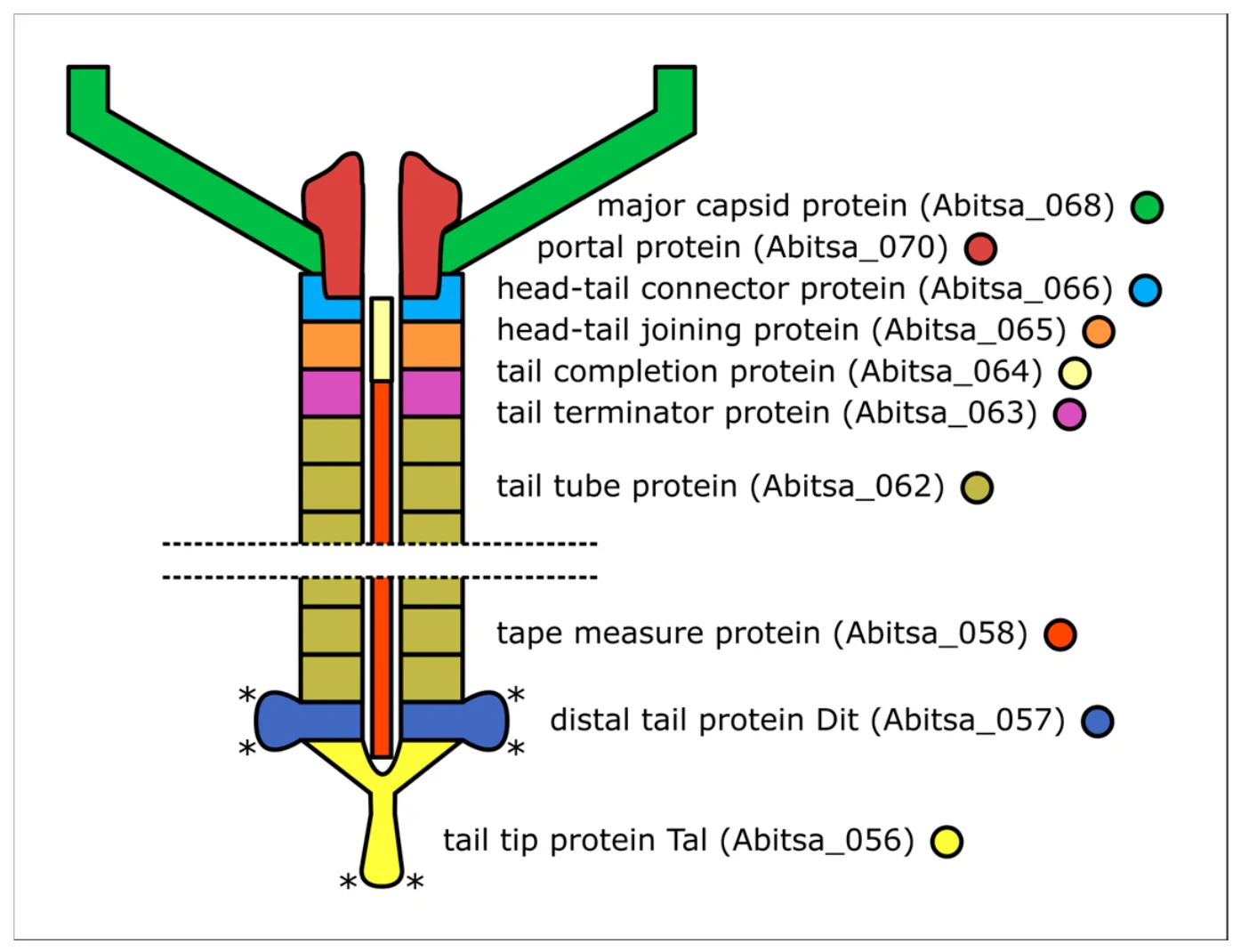
Putative minimal structure of the phage Abitsa tail and tail–head junction. Proteins are labeled according to their predicted functions. Asterisks denote putative receptor-binding sites.

The region spanning Abitsa_066-Abitsa_063 encodes phage neck proteins. As no universal nomenclature exists for these proteins, we followed the terminology used for *Staphylococcus* phage 80α, a siphovirus with a well-studied structure [58] that possesses homologs of all the corresponding proteins. This region contains consecutive genes encoding the head–tail connector protein (Abitsa_066), head–tail joining protein (Abitsa_065), tail completion protein (Abitsa_064), and tail terminator protein (Abitsa_063). The first two proteins form rings attached to the portal protein, whereas the latter two complete the tail structure; upon assembly, these components join to produce a phage tail and tail–head junction (Figure 7) [58,59]. The head–tail connector protein is known to be structurally diverse in siphoviruses, but is presumed to be evolutionarily related across the group [58]. In phage Abitsa, it consists of a bundle of alpha-helices similar to gp6 of *Escherichia* phage HK97 (PDB 3jvo) [60].

The genome contains the canonical module of the siphovirus tail initiator complex [61]: a triad of consecutive genes encoding the tape measure protein (TMP), distal tail protein (Dit), and tail tip protein (Tal). The C-terminal region of TMP (Abitsa_058, residues 1246–1370) corresponds to the conserved domain cd13402, a GH23-family lytic transglycosylase that cleaves the beta-1,4-glycosidic bond in peptidoglycan. This domain may become functionally exposed during TMP release and could facilitate cell wall penetration.

**Figure 8:**
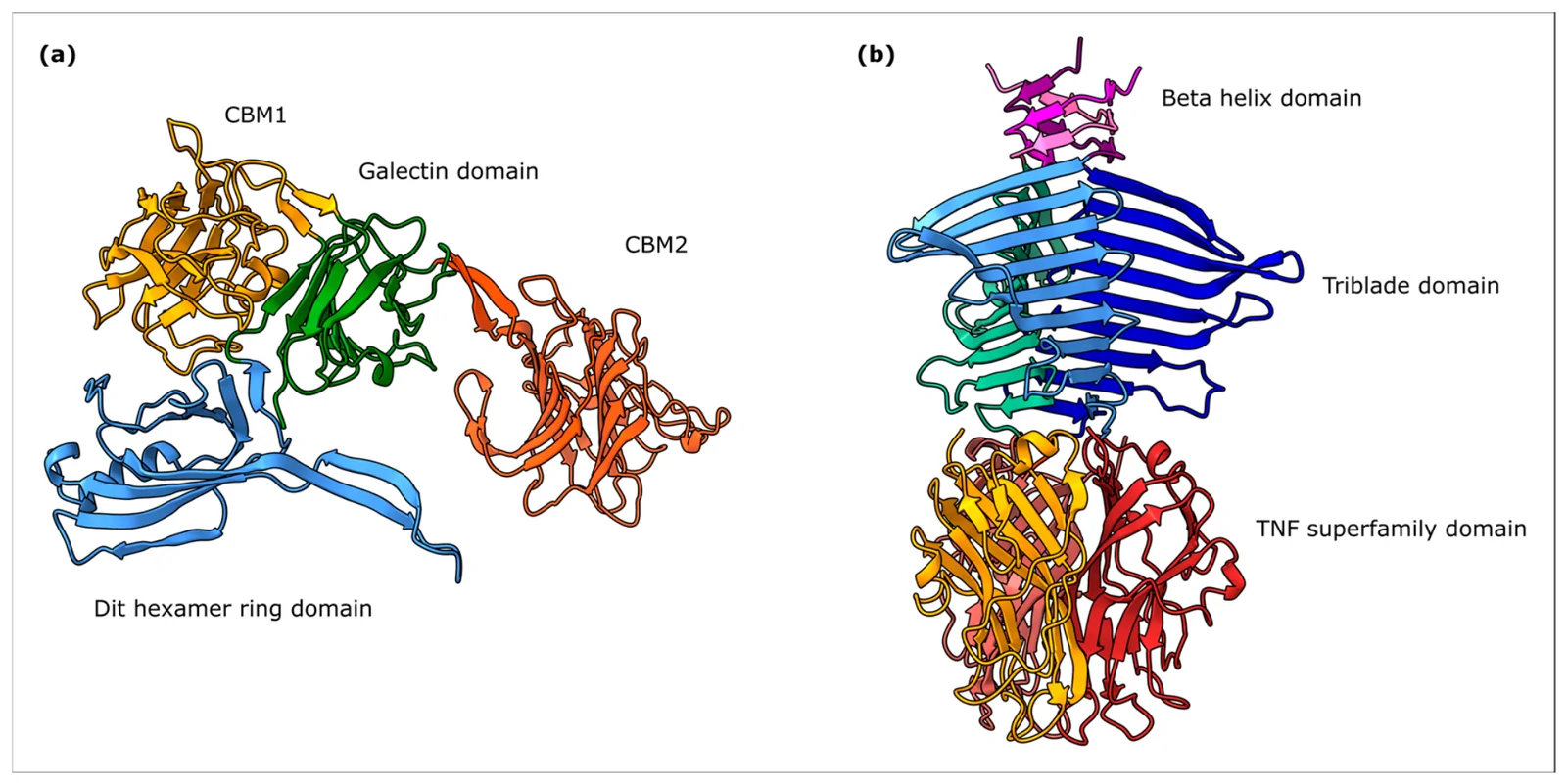
(**a**) AlphaFold3 predicted structure of distal tail protein (Dit) monomer. Galectin domain harbors two inserted carbohydrate binding modules (CBM). (**b**) AlphaFold3 predicted structure of C-terminal part of tail tip protein trimer.

The Dit protein (Abitsa_057), whose N-terminal part forms a hexameric ring onto which the tail tube proteins are assembled, is additionally functionalized, representing an “evolved Dit” structure [62]. Similarly to *Lactobacillus* phage J-1, it contains two insertions to a galectin domain (Figure 8a), forming carbohydrate binding modules (CBM).

The first one, termed CBM1 (residues 145–326), is structurally similar to multiple carbohydrate-binding domains, including CBMs from *Caldanaerobius polysaccharolyticus* endoglucanase (PDB 2zex), *Microbacterium arabinogalactanolyticum* endo-alpha-D-arabinofuranosidase EndoMA1 (PDB 8hhv), *Acetivibrio thermocellus* endo-1,4-beta-D-xylanase Xyn10B (PDB 2wze), *Acetivibrio thermocellus* cellulase CelK (PDB 3p6b) and *Thermotoga maritima* endo-β-1,4-galactanase (PDB 2xon). The predominant ligands for the close related proteins seems to be linear beta-1,4-linked glycans, suggesting them as a candidate receptor for CBM1 [63,64,65]. CBM1 is also structurally related to proteins from distant phage families including tail spike protein Tsp4 from *Escherichia* phage CBA120 (PDB 5w6h) and tail fiber gp52 from *Klebsiella* phage Kp7 (PDB 7xyc).

The second insertion into Dit, termed CBM2 (residues 375–620), is related to a CBM2 in Dit protein of *Lactobacillus* phage J-1 (PDB 5ly8), sharing 24.8% sequence identity and RMSD 2.56 over 98% query coverage. This domain was shown to bind a rhamnose-rich polysaccharide of *Lactobacillus casei* BL23 [62]. To a lesser extent, this domain is related to a variety of eukaryotic lectins including human ERGIC-53 (PDB 4ygb).

The N-terminal part of the Tal protein (Abitsa_056, residues 1–370) presumably forms a trimeric ring that attaches to the hexameric Dit ring and encloses the C-terminal alpha helices of TMP [66,67,68]. Its predicted structure in phage Abitsa is similar to the experimentally determined structures of siphovirus *Staphylococcus* phage 80α Tal (PDB 6v8i) as well as baseplate hub proteins of myoviruses *Escherichia* phage Mu (PDB 1wru) and *Shewanella* phage MuSo2 (PDB 3cdd). Unlike *Brussowvirus* phages, this N-terminal part is not functionalized by additional domains [69]. Following region of the protein (residues 371–450) putatively form a plug that seals tail channel and restructures following receptor binding, however, its structure and position cannot be robustly predicted using AlphaFol3 and Chai1.

The C-terminal part of the Tal protein (residues 451–742) forms a trimeric beta-sheet stem, consisting of a predicted short beta-helix followed by a trimer of large beta-sheets, which form three elongated blades (Figure 8b). Each of these blades has a path of negatively charged residues on the inner edge (Glu469, Asp491, Asp522, Asp458, Glu573) and a path of positively charged residues on the side (Lys483, Lys512, Arg540, Lys563). These paths form an interface between neighboring subunits with high electrostatic complementarity, assembling them in a triblade structure. At the tip, Tal has a trimeric TNF superfamily domain, structurally related to a wide variety of experimentally determined structures including human ectodysplasin A (PDB 1rj7), C1q globular head (PDB 2wnv), *Bacillus anthracis* exosporium protein BxpB (PDB 8d02) and *Helicobacter* phage KHP30 cement protein (PDB 7dn2). As the related domains are involved in protein–protein interactions, we suppose this terminal TNF superfamily domain may act as a receptor-binding domain. Notably, a putative solvent-accessible hydrophobic patch is formed in the cleft between subunits, consisting of side chains of Tyr659, Tyr661, Tyr681 and Tyr688, constituting a candidate interaction surface.

### 3.8. Intergenomic Similarity Comparisons and Phylogenetic Analysis

**Figure 9:**
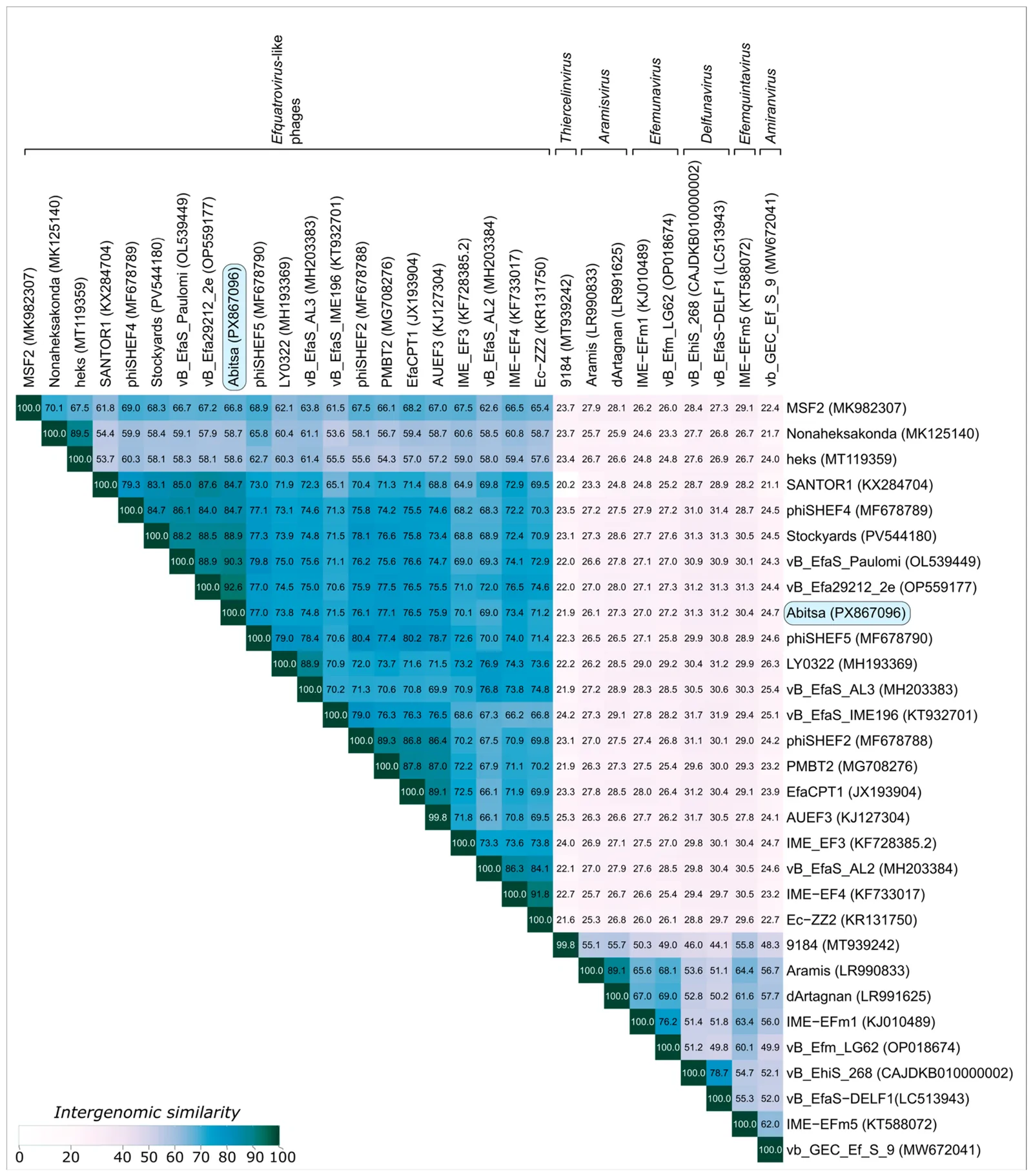
VIRIDIC heatmap generated using genomic sequences of the related *Enterococcus*-infecting phages. Clusters corresponding to the genera classified by ICTV Virus Taxonomy 2024 Release are denoted at the top.

A BLASTn search against the core_nt database, together with VIRIDIC analysis (Figure 9), showed that the closest related phage to Abitsa is *Enterococcus* phage vB_Efa29212_2e, with 92.6% intergenomic similarity. Among ICTV exemplar phages for virus species, the closest relatives are *Enterococcus* phages SANTOR1 and phiSHEF4, members of the genus *Efquatrovirus*, each showing 84.7% similarity. As these values fall below the proposed 95% threshold for phage species delineation, *Enterococcus* phage Abitsa represents a novel species within the genus *Efquatrovirus* under current guidelines [70].

Generally, VIRIDIC analysis reveals that the genus *Efquatrovirus* is highly heterogenous, poorly compatible to 70% genus threshold with multiple borderline cases; and a linage comprising phages heks and Nonaheksakonda was even proposed as separate genus not currently included in ICTV taxonomy [71]. This group is referred to hereafter as *Efquatrovirus*-like phages (EVL phages), because its members share a similar genomic architecture and form a coherent cluster with intergenomic similarity values generally >50% within the cluster and >58% relative to IME-EF4. However, for some of these phages, formal taxonomic assignment under a strict 70% genus threshold is not straightforward without introducing an excessive number of additional genera (Supplementary Figures S1–S4).

The search of additional Enterococcus-related phages with non-assigned taxonomic position revealed *Enterococcus* phages EFAP-1 [72] and EFRM31, which show lower VIRIDIC similarity to IME-EF4, at approximately 46–47%. Notably, the genomes of EFAP-1 and EFRM31 are approximately twofold shorter than those of typical efquatroviruses. In EFAP-1, the replication, recombination, and metabolism region present in Abitsa is absent. *Enterococcus* phage EFRM31 was reported to possess a head length of 62 nm, a head width of 55 nm, and a tail length of 206 nm [73]. These dimensions appear unusual for a phage with a genome size of only 16,945 bp and a tape measure protein of 125 aa. This discrepancy suggests either that the assemblies of EFRM31 and EFAP-1 require additional validation or that these phages represent unusual biological variants deserving dedicated investigation. Overall, if these assemblies are correct, one possible evolutionary interpretation is that the common ancestor of efquatroviruses, EFAP-1, and EFRM31 was a small siphovirus. In this scenario, one lineage acquired a replication module oriented oppositely to the structural module, as observed in Abitsa and related efquatroviruses, whereas another lineage retained or secondarily lost this module, resulting in the short genomes of EFAP-1 and EFRM31.

**Figure 10:**
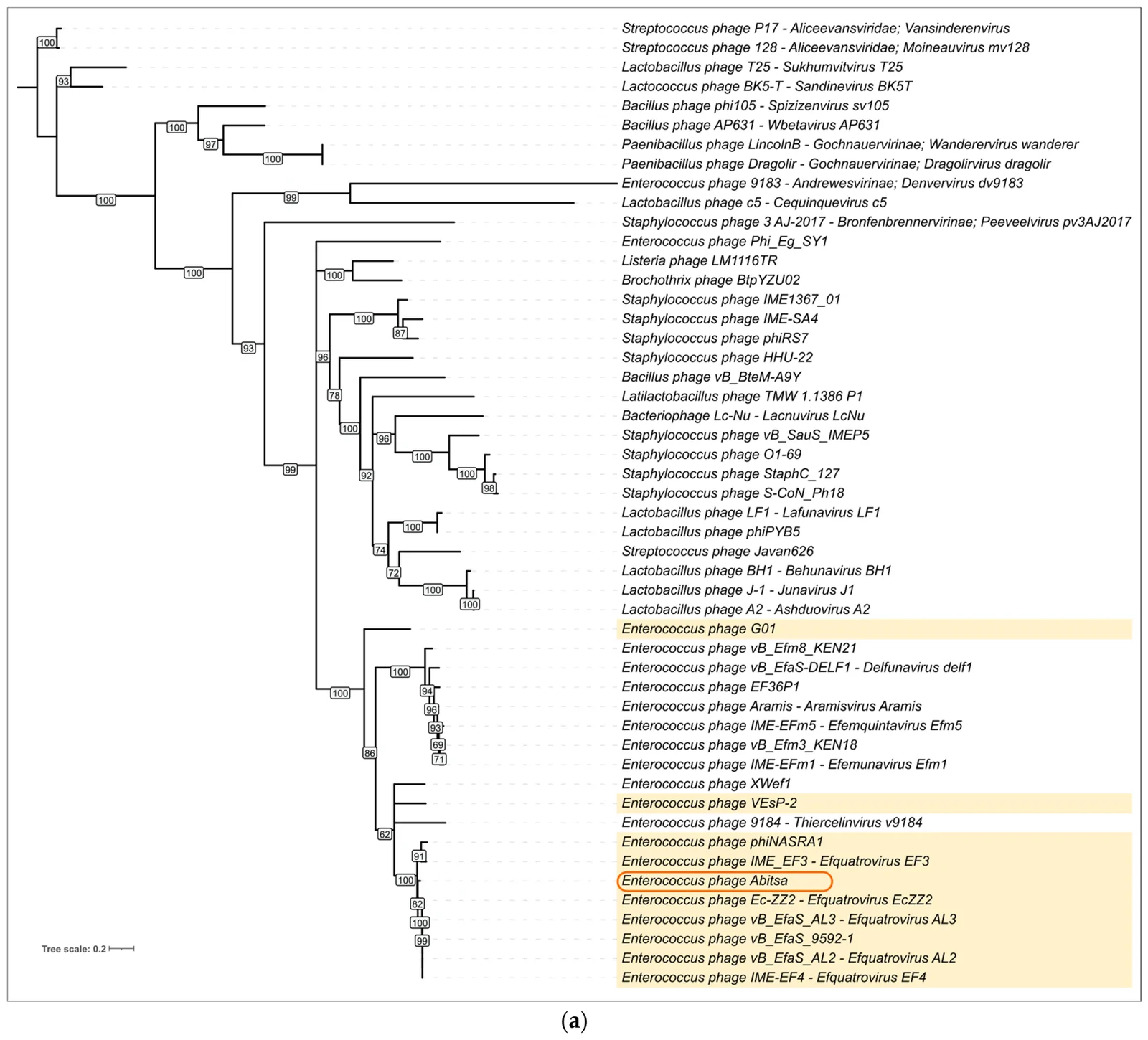

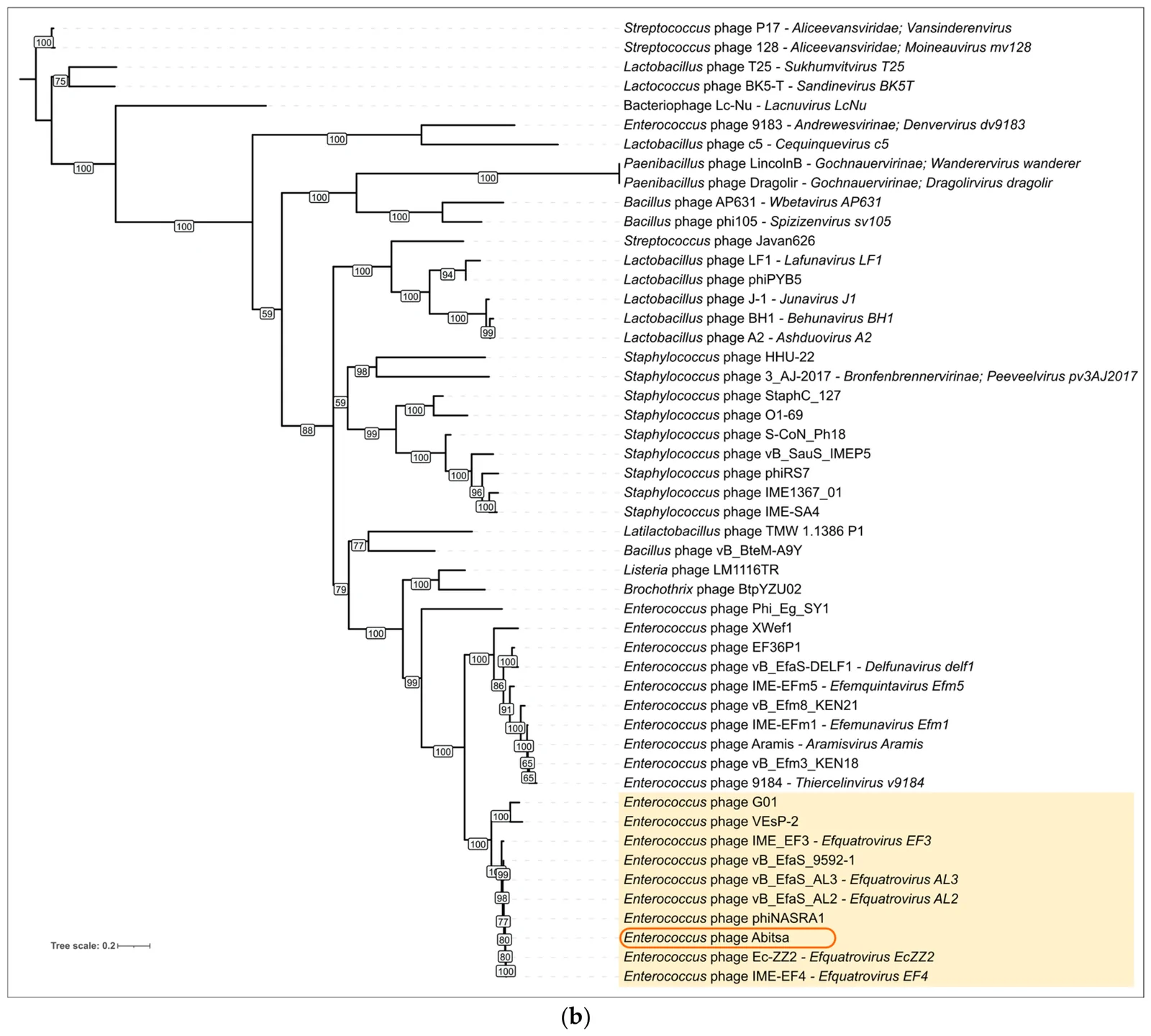
Phylogenetic tree inferred from 50 representative amino acid sequences of the (**a**) major capsid protein (MCP) and (**b**) terminase large subunit (TLS). Taxonomic assignments are shown in the tip labels and color-shaded blocks in the legend; binomial genus–species names are used for ICTV-classified phages, whereas NCBI annotations are used for unclassified phages. Phage Abitsa is highlighted with an orange rounded box. The group of phages that can be classified as *Efquatrovirus* is highlited in yellow. Bootstrap support values are shown next to the corresponding nodes. The scale bar indicates the expected number of substitutions per site, and the trees are rooted to Streptococcus phages P17 and 128.

Phylogenetic analysis was performed using representative 50-sequence datasets for the MCP, TLS, portal protein, major tail protein (tail tube protein), DNA helicase, DNA polymerase, distal tail protein, endolysin, and tail tip protein (Figure 10, Supplementary Figures S5–S11), as described in Section 2.11. In all trees, Abitsa was consistently placed within the clade containing efquatroviruses or their closest relatives, in agreement with the VIRIDIC-based classification. At the same time, the relative positions of individual EVL phages varied among marker proteins, indicating that internal relationships within this lineage are not fully congruent across genes. Such topological discordance is consistent with mosaic evolution and likely reflects past genetic exchange among EVL phages and closely related enterococcal phages.

### 3.9. Domain Shuffling Within Efquatrovirus

**Figure 11:**
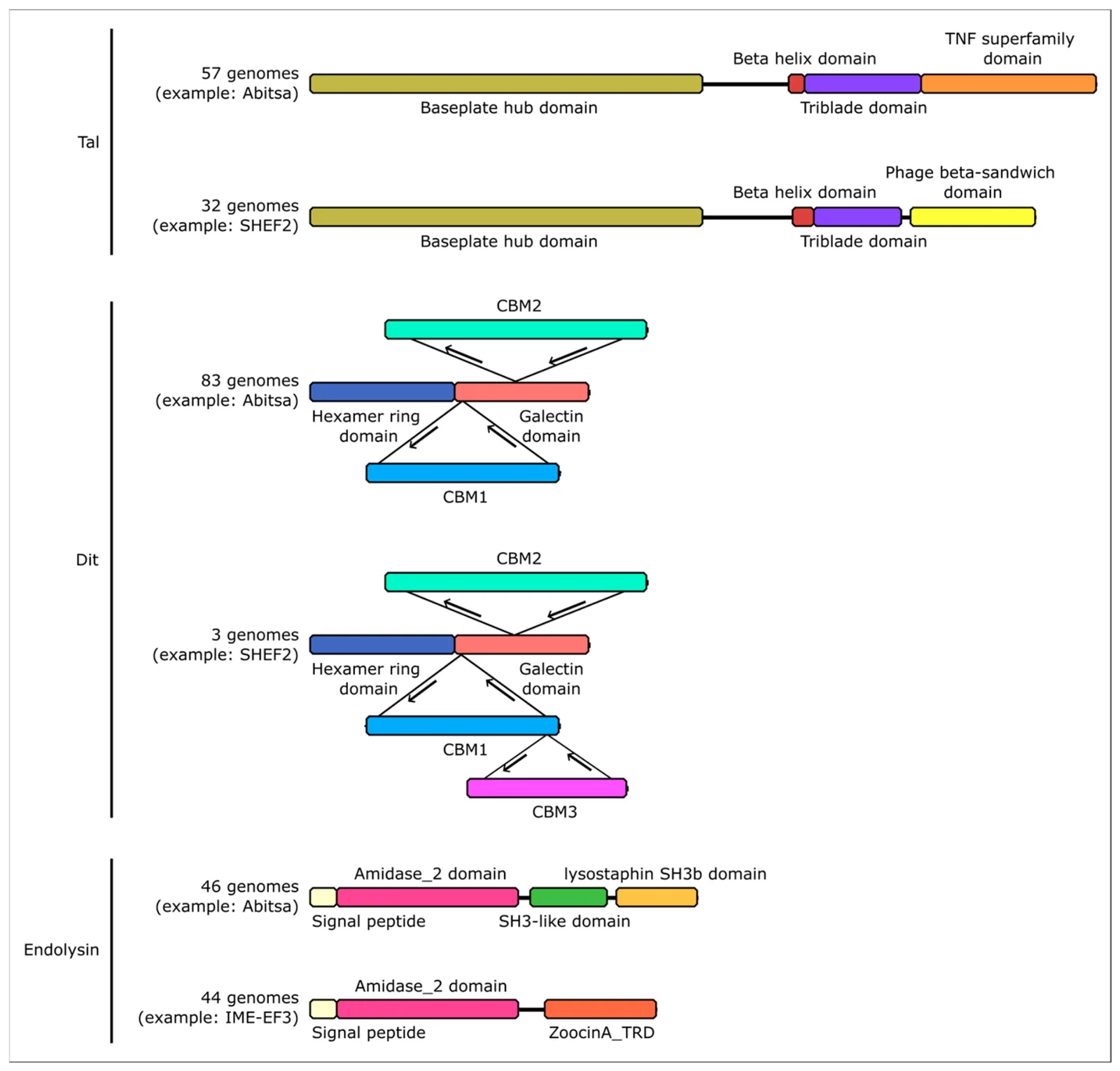
Domain folds of *Efquatrovirus*-like phages proteins, demonstrating domain shuffling. Direction from left to right corresponds to domain order from N-terminus to C-terminus. Dit proteins contain carbohydrate-binding modules CBM1 and CBM2 inserted into loops within the galectin domain, and may contain CBM3 inserted into a loop of CBM1. Different domains are shown in different colors, arrows denote intra-domain insertions.

The VIRIDIC analysis presented above highlights the substantial genomic diversity within *Efquatrovirus*, with multiple pairwise comparisons falling below the commonly used 70% genus-level similarity threshold. This diversity is explained not only by vertically inherited substitutions and indels, but also by horizontal gene transfer, including non-homologous replacement of domains in both structural and non-structural proteins. To assess the extent of this domain shuffling, we carried out a comparative genomic analysis (Figure 11) of 90 EVL genomes in addition to Abitsa, including distant relatives of currently undefined taxonomic status, such as MSF2, G01, heks, and Nonaheksakonda.

Of 91 genome sequences, 89 encode non-disrupted Tal proteins. In phage Abitsa protein contains C-terminal TNF superfamily domain as putative receptor binding domain as shown above, and this structure is shared with 56 other phages. In contrast, 32 *Efquatrovirus* phages including SHEF2, AUEF3 and PMBT2, encoded an unrelated C-terminal beta-sandwich domain. AlphaFold3 predicted structure (Supplementary Figure S12) shared similar fold with C-terminal domains of *Staphylococcus* phage K receptor-binding protein gp144 (PDB 5m9f) and *Escherichia* phage T7 gp17 tail fiber (PDB 4a0u), as well as the non-FhuA-binding subdomain of *Escherichia* phage T5 pb5 (PDB 8b14). The observed C-terminal diversification of *Efquatrovirus* Tal provides additional evidence for its role as a determinant of the host range.

Full-length Dit protein is found in 86 EVL genome sequences. Nearly all of them, including protein of phage Abitsa, share the same fold with hexamer ring domain and galectin domain with two CBM inserted into loops of the latter. However, phages IME-EF3, EFM3116TR and MSF2 harbor even more “evolved” Dit with an additional CBM3 inserted in C-terminal loop of CBM1; this domain itself is structurally similar to CBM1 (RMSD 3.04 over 83% query coverage).

A total of 90 EVL genomes encode non-fragmented endolysin proteins. While N-terminal Amidase_2 domain is highly conserved, there are two distinct types of C-terminal parts with nearly equal prevalence. A total of 46 phages, including Abitsa, have SH3-like and lysostaphin SH3b domains, while 44 phages carry instead a single unrelated ZoocinA_TRD domain which has been previously discovered in peptidoglycan hydrolyzing enzymes but its function remains undescribed. This pattern suggests recurrent exchange of putative cell-wall-binding modules to bind different peptidoglycan structures while preserving the conserved catalytic core.

Noteworthy, the observed domain organization divergence and general phylogeny in these three proteins was not associated with overall genome sequence identity or phylogenetic trees of the most conserved phage proteins. E.g., MSF2 and IME-EF3 phages have the same CBM3 insertion in Dit despite only 67.5% VIRIDIC intergenomic similarity.

## 4. Discussion

### 4.1. Biological Properties, Host Range and Therapeutic Potential of Phage Abitsa

The optimal multiplicity of infection for phage Abitsa was 0.01, indicating a high lytic activity at low infection ratios, and implying that a low initial dose could achieve therapeutic effects [74].

The bacterial growth inhibition assay showed that phage Abitsa effectively suppressed planktonic growth of *E. faecalis*, with higher MOIs producing more rapid and prolonged inhibition. Following the initial lysis phase, bacterial regrowth was observed in all phage-treated cultures. Resistant colonies were detected from 4 h onward at MOIs of 10, 1, 0.1, and 0.01, and from 6 h onward at an MOI of 0.001. These results indicate that the later increase in OD600 was associated with the expansion of a phage-resistant subpopulation [75,76].

The data obtained in this study demonstrate that the bacteriophage Abitsa possesses a significant and statistically valid capability to disrupt *E. faecalis* biofilms (*p* = 0.005), as confirmed by Tukey’s test.

While significant biofilm reduction was observed at the higher MOIs (10 and 1), the most pronounced and consistent antibiofilm activity occurred at the lower concentrations of 0.1 and 0.01. This phenomenon, where lower concentrations yield greater efficacy, could be explained by several biological mechanisms. It is possible that at very high concentrations, phage particles may aggregate or experience reduced diffusion within the biofilm matrix, thereby limiting their access to target cells.

The one-step curve of phage Abitsa was similar to the one observed for closely related phage vB_Efa29212_2e [77]. Both phages exhibit short latent period and rapid increase in phage titer up to 1 × 10^10^ after only 70 min of incubation, which reflects the cumulative effect of several replication cycles. Although Abitsa demonstrated shorter latent period of ten minutes and less progeny phages than vB_Efa29212_2e, only 28 ± 5. Stability in a wide range of pH and temperature levels shows that the phage can maintain its activity in oral cavity and root canals. Described growth characteristics and stability in different environmental conditions are an advantage for therapy, however, the demonstrated narrow host range greatly limits its potential and in vitro directed evolution based on the “Appelmans protocol” [78] might be needed to use this phage in a therapeutic cocktail in the future. This specificity is not uncommon for this genus, as another *Efquatrovirus* phage vB_EfaS_LOK1 was also found to have a limited host range, lysing only one *E. faecalis* strain used for phage isolation [79].

Although the antibiofilm activity and environmental stability of phage Abitsa are consistent with potential application in endodontic infections, direct evaluation on dental substrates such as dentin discs or in root canal models was beyond the scope of this study and represents an important direction for future investigation.

### 4.2. Genome Features of Phage Abitsa

Comparative analysis of intergenomic similarity and single-protein phylogenies consistently places *Enterococcus* phage Abitsa as a novel species within the genus *Efquatrovirus*, which is currently not assigned to any family or subfamily by ICTV. At the same time, the partial incongruence among individual protein trees supports a role for genetic exchange in the evolution of Abitsa and related *Efquatrovirus*-like phages, indicating that the Abitsa genome was shaped not only by vertical divergence but also by mosaic processes affecting particular genes and modules.

Phage Abitsa may employ a capsid assembly strategy similar to *Escherichia* phages HK97 and T5 [55,80,81], as its major capsid protein is fused to an N-terminal scaffolding region which is later removed during maturation. Consistent with this organization, the accompanying Abitsa capsid maturation protease belongs to family S78, the same family as HK97 gp4 and T5 pb11. In both of these phages, the proteases cleave substrates after lysine residues [80,81]; therefore, if this substrate specificity is conserved in Abitsa, the high density of lysine residues within the putative scaffolding region could generate multiple protease recognition sites and facilitate scaffold removal during prohead maturation.

In a study of *Efquatrovirus* SHEF2, both adsorption to *Enterococcus* cells and successful infection were shown to be affected by different mutations in genes involved in enterococcal polysaccharide antigen biosynthesis, implicating both the core polysaccharide and its decoration residues as potential receptors [25]. Another study revealed that an *E. faecalis* strain resistant to *Efquatrovirus* EFap02 had loss-of-function mutations in glycosyltransferase, resulting in capsule loss [75]. Together, these findings are consistent with our identification of carbohydrate-binding modules in the Dit protein of phage Abitsa as putative receptor-binding domains. However, there is no compelling evidence that surface glycans are the only receptors involved in Abitsa infection.

According to a proposed model, receptor binding proteins of siphophages can typically be categorized into two groups: host-recognition receptors facilitating adsorption and correct phage orientation, and membrane-sensing receptors that recognize membrane elements and trigger tube opening [82]. Within this framework, the carbo-hydrate-binding modules of Dit could either perform both functions or participate only in reversible adsorption, whereas the C-terminal region of Tal may detect membrane adjacency. This interpretation is supported by the mutagenesis study cited above, in which some enterococcal polysaccharide biosynthesis mutants still permitted SHEF2 surface binding but failed to support productive infection [25]. The same study also showed that the C-terminal regions of both Dit and Tal are highly divergent among the *Efquatrovirus* species SHEF2, SHEF4, and SHEF5, leading the authors to propose that these regions are key determinants of host range [25]. Our results reinforce this statement by showing that *Efquatrovirus* species may possess not merely highly divergent, but even unrelated, folds at the very tip of Tal as well as insertions of an additional CBM in Dit. Such domain shuffling is widespread in receptor-binding proteins, allowing rapid adaptation to new host lineages [17].

Mosaicism within protein sequences is not limited to adsorption machinery but extends to the lysis module. A previous study has also demonstrated domain shuffling in endolysins of *Enterococcus* phages [83], putatively reflecting a potential to degrade different structures or arrangements of peptidoglycan chains [17]. Our analysis demonstrate that the two distinct domain organizations of this protein is wide-spread within the same genus *Efquatrovirus*, additionally underscoring its genetic diversity and adaptations to particular subsets of host strains.

## 5. Conclusions

In this work, we isolated and comprehensively characterized *Enterococcus* phage Abitsa, a lytic siphovirus representing a novel species within the genus *Efquatrovirus*. Phenotypic assays and antibiofilm experiments showed that Abitsa has high lytic activity, pronounced antibiofilm potential and a narrow host range. The rapid development of bacterial resistance to this phage observed in planktonic culture, may be a limiting factor for its use alone. However, creating a phage cocktail based on phages with different receptors will suppress the growth of resistant bacteria. Given its high efficacy against biofilms, Abitsa phage could be an important addition to the phage cocktail. Overall, these results expand current knowledge of *Efquatrovirus* diversity and indicate that Abitsa may be considered a candidate for further development as a component of personalized phage cocktails against *Enterococcus faecalis*.

## Figures and Tables

**Table 1:** Determination of host range of phage Abitsa.

Isolate ID	Species	Source	Lysis
ATCC19433	*E. faecalis*	American type culture collection	+
2510	*E. faecalis*	Collection of IBCh RAS	-
LT1485	*E. faecalis*	Collection of Gabrichevsky institute of epidemiology and microbiology	-
LT6B7F	*E. faecalis*		+
LT6B7B	*E. faecalis*		-
LT6B80	*E. faecalis*		-
LT6B7E	*E. faecalis*		-
LT6B7A	*E. faecalis*		-
77046	*E. faecalis*		-
77047.2	*E. faecalis*		-
770111	*E. faecalis*		-
71	*E. faecalis*		-
13539	*E. faecalis*		-
76.1	*E. faecalis*		-
77044	*E. faecalis*		-
77112	*E. faecalis*		-
770114	*E. faecalis*		-
10	*E. faecalis*		-
LT6B83	*E. faecium*		-
LT1493	*E. faecium*		-
LT6B85	*E. faecium*		-
LT6B89	*E. faecium*		-
137	*E. faecalis*	Cerebrospinal fluid	-
140	*E. faecalis*	Phlegmon	-
181	*E. faecalis*	Root canal	-
202	*E. faecalis*		-
203	*E. faecalis*		-
213	*E. faecalis*		-
214	*E. faecalis*		-
206	*E. faecalis*		-
62	*E. faecium*		-
63	*E. faecium*		-
115	*E. faecium*		-
116	*E. faecium*		-

+: Lytic activity, -: No lytic activity (no plaques).

## Data Availability

All relevant data are available within this article and its Supplementary Materials. The Enterococcus phage Abitsa genome sequence is deposited in NCBI GenBank under accession number PX867096.1.

## Supplementary Materials

The following supporting information can be downloaded at: https://www.mdpi.com/article/10.3390/v18080842/s1, Figure S1: VIRIDIC heatmap showing pairwise intergenomic similarities among 112 Abitsa-related phages identified by BLAST search of the GB PHG database using the Abitsa major capsid protein sequence. Figure S2: Distribution of pairwise VIRIDIC intergenomic similarity values relative to *Enterococcus* phage IME-EF4 among the analyzed Abitsa-related phages. Figure S3: Empirical cumulative distribution function (ECDF) of pairwise VIRIDIC intergenomic similarity values relative to *Enterococcus* phage IME-EF4 among the analyzed Abitsa-related phages. Figure S4: Group composition across 5% bins of pairwise VIRIDIC intergenomic similarity values relative to *Enterococcus* phage IME-EF4 among the analyzed Abitsa-related phages. Figure S5: Phylogenetic tree inferred from 50 representative amino acid sequences of the portal protein. Figure S6: Phylogenetic tree inferred from 50 representative amino acid sequences of the major tail (tail tube) protein (MTP). Figure S7: Phylogenetic tree inferred from 50 representative DNA helicase protein sequences. Figure S8: Phylogenetic tree inferred from 50 representative DNA polymerase protein sequences. Figure S9: Phylogenetic tree inferred from 50 representative amino acid sequences of the distal tail protein. Figure S10: Phylogenetic tree inferred from 50 representative endolysin protein sequences. Figure S11: Phylogenetic tree inferred from 50 representative amino acid sequences of the tail tip protein (Tal). Figure S12: AlphaFold3 predicted structures of C-terminal parts of the tail tip proteins (Tal) of Abitsa and SHEF2.

## Abbreviations

The following abbreviations are used in this manuscript:

BHI	Brain heart infusion
bp	Base pair
CBM	Carbohydrate binding modules
CFU	Colony-forming units
gp	Gene product
ICTV	International Committee on Taxonomy of Viruses
MCP	Major capsid protein
MOI	Multiplicity of infection
NCBI	National center for biotechnology information
OD	Optical density
PFU	Plaque-forming units
TLS	Terminase large subunit
TMP	Tape measure protein
